# Nuclear Radiation Shielding Characteristics of Some Natural Rocks by Using EPICS2017 Library

**DOI:** 10.3390/ma14164669

**Published:** 2021-08-19

**Authors:** Mohammed Sultan Al-Buriahi, M. I. Sayyed, Rashad A. R. Bantan, Yas Al-Hadeethi

**Affiliations:** 1 Physics Department, Sakarya University, Sakarya 54050, Turkey; mohammed.al-buriahi@ogr.sakarya.edu.tr; 2 Department of Physics, Faculty of Science, Isra University, Amman 11622, Jordan; mabualssayed@ut.edu.sa; 3 Department of Nuclear Medicine Research, Institute for Research and Medical Consultations (IRMC), Imam Abdulrahman bin Faisal University (IAU), P.O. Box 1982, Dammam 31441, Saudi Arabia; 4 Department of Marine Geology, Faculty of Marine Science, King Abdulaziz University, Jeddah 21589, Saudi Arabia; rbantan@kau.edu.sa; 5 Physics Department, Faculty of Science, King Abdulaziz University, Jeddah 21589, Saudi Arabia; 6 Lithography in Devices Fabrication and Development Research Group, King Abdulaziz University, Jeddah 21589, Saudi Arabia

**Keywords:** radiation, shielding, EPICS2017 library, rock

## Abstract

Radiation leakage is a serious problem in various technological applications. In this paper, radiation shielding characteristics of some natural rocks are elucidated. Mass attenuation coefficients (µ/ρ) of these rocks are obtained at different photon energies with the help of the EPICS2017 library. The obtained µ/ρ values are confirmed via the theoretical XCOM program by determining the correlation factor and relative deviation between both of these methods. Then, effective atomic number (Z_eff_), absorption length (MFP), and half value layer (HVL) are evaluated by applying the µ/ρ values. The maximum μ/ρ values of the natural rocks were observed at 0.37 MeV. At this energy, the Z_eff_ values of the natural rocks were 16.23, 16.97, 17.28, 10.43, and 16.65 for olivine basalt, jet black granite, limestone, sandstone, and dolerite, respectively. It is noted that the radiation shielding features of the selected natural rocks are higher than that of conventional concrete and comparable with those of commercial glasses. Therefore, the present rocks can be used in various radiation shielding applications, and they have many advantages for being clean and low-cost products. In addition, we found that the EPICS2017 library is useful in determining the radiation shielding parameters for the rocks and may be used for further calculations for other rocks and construction building materials.

## 1. Introduction

Radiation is around us all the time. Everyone on the planet is getting irradiated every day because radiation comes from the sun, ground, and from different man-made sources. Nowadays, ionizing radiation is used in a wide variety of fields, such as nuclear power, manufacturing, research, and medicine, as well as many other areas [1,2]. However, it presents a health hazard if proper measures are not followed against undesired exposure. For example, exposure to such radiation causes great damage to the human being and the surrounding environment [3,4]. Lead (Pb) and conventional shielding materials (e.g., concrete) are the most common materials utilized to block the damaging radiation in various applications [5,6,7]. Such materials are cheap, abundant, and valid to absorb the damaging radiation [8,9]. However, Pb-based materials have their own associated health hazards [10,11,12,13,14,15]. Therefore, it is important to search for reliable, clean, and inexpensive alternative candidates to void the effects of damaging radiation [16,17,18,19,20,21,22,23,24,25].

Rocks are a part of what can be seen everywhere and every day. They are inexpensive and can be useful for many applications. For example, limestone is used for cement, bituminous coal is used for electric power. This triggered many authors to study the photon shielding properties of some natural rock; for example, Obaid et al. determined gamma shielding features of rocks and concrete [10]. It was found that feldspathic basalt, volcanic rock, compact basalt, pink granite, and dolerite were better than concrete for attenuating gamma-rays. Agar et al., introduced an experimental investigation to test the photon attenuation for some concretes [17]. Waly and Bourham compared different types of concretes as shielding materials against gamma-rays [23]. The previous studies showed that the radiation shielding characteristics of any material can be described by several parameters, like μ/ρ, HVL, Z_eff_ and MFP [1,6,7,10].

The current work aims to study the radiation shielding features of some natural rocks, including olivine basalt, jet black granite, limestone, sandstone, and dolerite. The radiation shielding characteristics (μ/ρ, HVL, Z_eff_ and MFP) of these rocks were investigated via the EPICS2017 library. The calculated μ/ρ values were determined via the EPICS2017 library, confirmed using XCOM and then utilized to obtain all the important radiation shielding parameters. Shielding characteristics of the selected rocks were compared to those of conventional concrete and commercial glasses. The present work introduced a superior and environment-friendly alternative for radiation shielding applications.

## 2. Materials and Method

Some natural rocks such as olivine basalt, jet black granite, limestone, sandstone, and dolerite were tested in terms of nuclear shielding efficiency. The weight fraction of the elements in these rocks along with their densities are given in Table 1. The nuclear shielding efficiency of the tested rocks was investigated by using the EPICS2017 library. EPICS2017 was found to be a useful method for the evaluation of the mass attenuation coefficients for different materials [26,27,28,29,30]. Previously, Obaid et al. [26] used Monte Carlo simulation via MCNPX and Geant4 to report the μ/ρ values and other parameters for some of the investigated rocks in this work. Moreover, Obaid et al. [6,7] used an experimental method to report the radiation attenuation factors for some of the investigated rocks in this study. The novelty in this work is that we used the same rocks reported in [6,7,26], but we used another technique, namely the EPICS2017 library, and reported the μ/ρ values and other factors. The aim with this work was to check the possibility of using the EPICS2017 library to calculate the μ/ρ, HVL, etc., for some rock samples, and thus to find an alternative technique to evaluate the radiation attenuation abilities for any rocks. This will help other researchers find an effective technique to study the radiation shielding parameters for other rocks in the absence of the necessary equipment to carry out the practical part.

In the current investigation, we used the EPICS2017 library to calculate the radiation through some chosen rocks at energies between 0.365 and 2.510 MeV. Moreover, we used the theoretical calculations via XCOM to confirm the accuracy of our outcomes.

In addition, the other shielding parameters such as half value layer (HVL) and effective atomic number (Z_eff_) were computed through the help of the Phy-X/PSD software [31]. The details of calculation procedures for the radiation shielding parameters can be found in [32,33,34,35,36,37,38,39].

## 3. Results and Discussion

Table 1 displays the rock name, atomic composition, and density for the natural rocks under study. The radiation shielding characteristics of these rocks were tested by using the EPICS2017 library. Firstly, the µ/ρ of these rocks were obtained at the 0.37–2.51 MeV region using the EPICS2017 library. The EPICS2017-obtained results were validated by the theoretical values of XCOM as shown in Table 2 and Table 3. The deviation (Dev%) values were calculated using the following equation:

(1) $$Dev.=\frac{{\left(\mu /\rho \right)}_{\mathrm{EPICS}2017-{\left(\mu /\rho \right)}_{\mathrm{XCOM}}}}{{\left(\mu /\rho \right)}_{\mathrm{XCOM}}}\times 100\%\;\;\;$$

**Figure 1:**
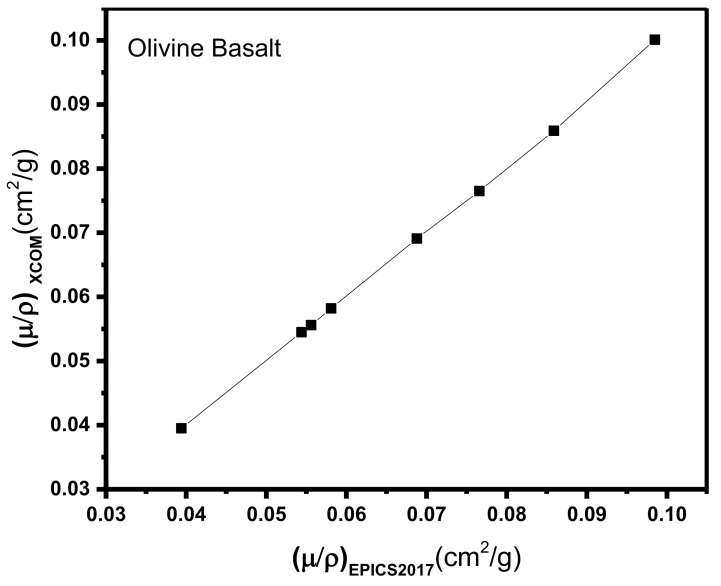
The validation of EPICS2017 by using XCOM calculations.

**Figure 2:**
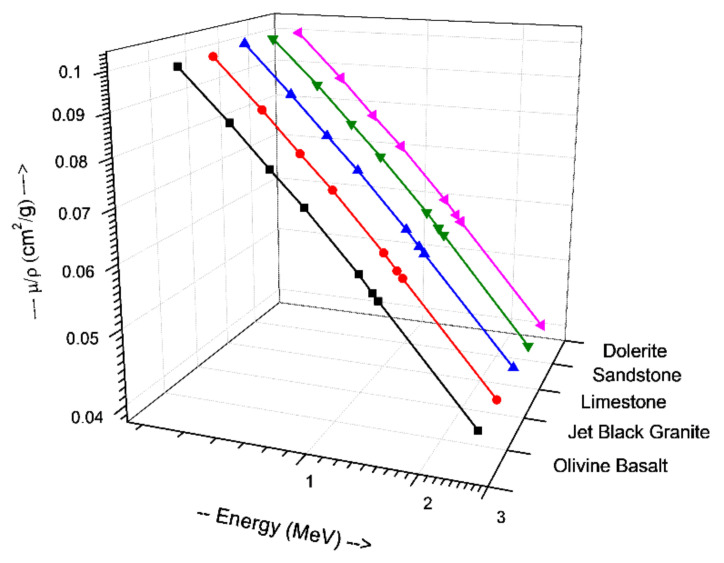
Mass attenuation coefficient (μ/ρ) obtained by EPICS2017 of the natural rocks at the 0.37–2.51 MeV region.

**Figure 3:**
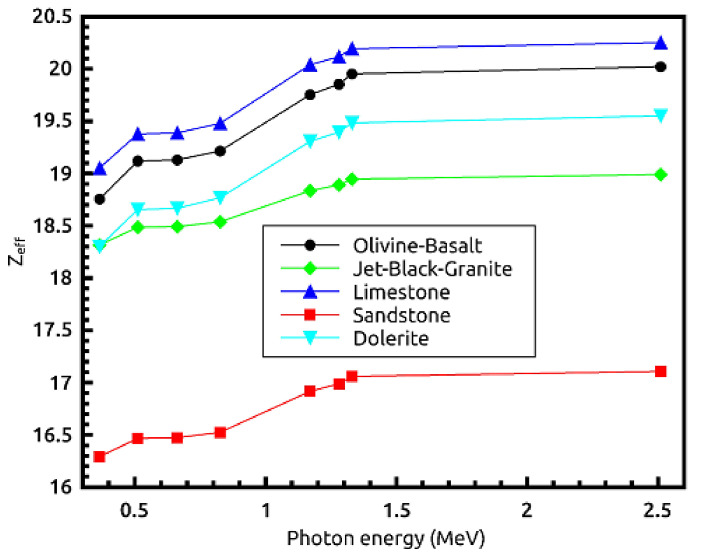
Effective atomic number (Z_eff_) of the natural rocks at the 0.37–2.51 MeV region.

**Figure 4:**
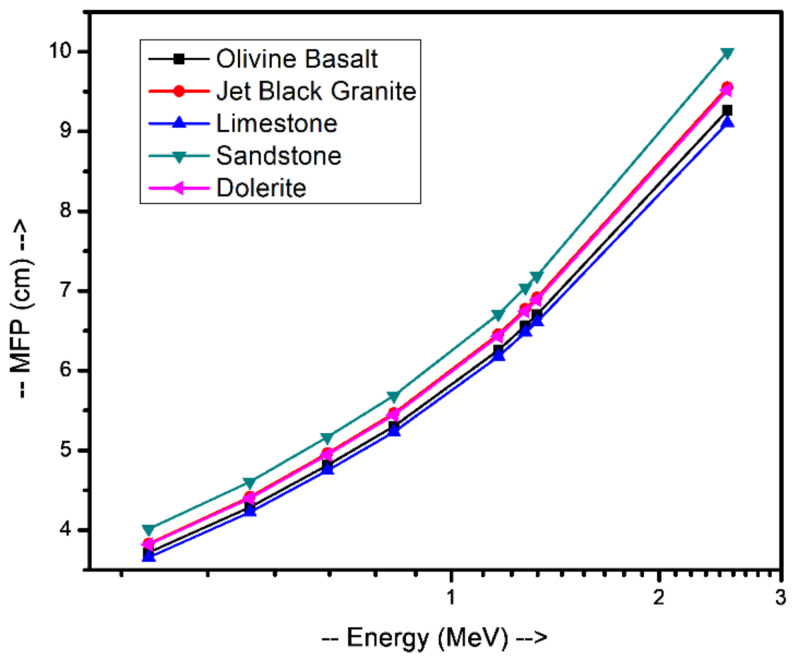
Mean free path (MFP) of the natural rocks at the 0.37–2.51 MeV region.

**Figure 5:**
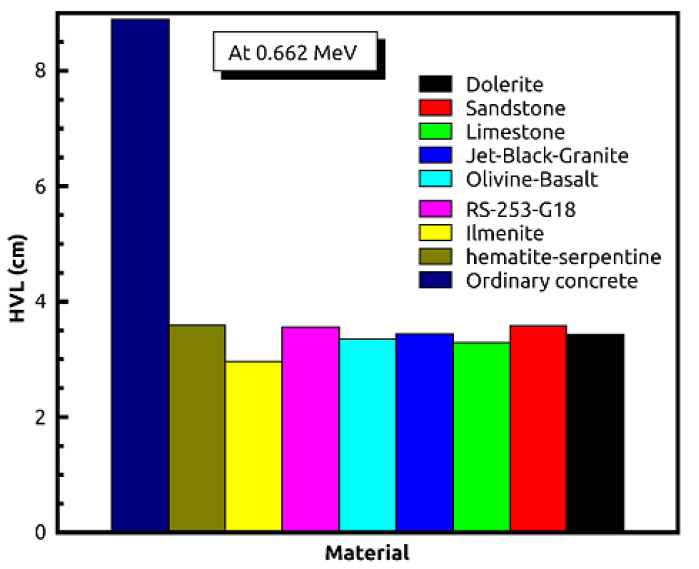
Comparison of the natural rocks with common shielding materials in terms of HVL at 0.662 MeV.

**Figure 6:**
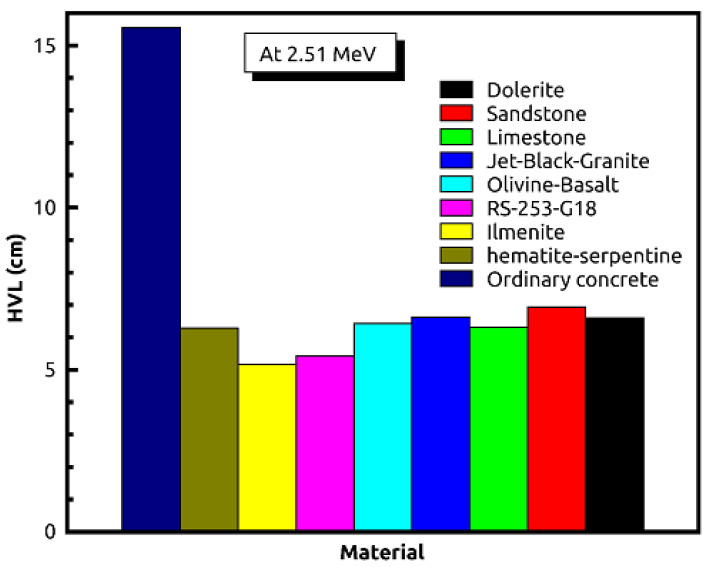
Comparison of the natural rocks with common shielding materials in terms of HVL at 2.51 MeV.

Table 2 and Table 3 show that the results of EPICS2017 are very close to the values of XCOM. The highest Dev% is noted around 2%. Moreover, Figure 1 displays a correlation factor between the EPICS2017 and XCOM µ/ρ in the case of olivine basalt. Clearly, the correlation factor is almost one for the photon energies in the region of 0.37–2.51 MeV. Figure 2 displays the energy dependence of the µ/ρ values of the selected natural rock. One may see that the µ/ρ values of the selected rocks are very close to each other, such that they are in the range of 0.0395–0.1001 cm^2^∙g^−1^. The Z_eff_ values of the rocks were calculated, and the results can be seen in Figure 3. The Z_eff_ values of the studied natural rock were in the range of 16 and 21. The maximum values of Z_eff_ were noted for limestone rock, because limestone rock contains the highest concentration of Ca (Z = 20, relative high-Z element). Therefore, limestone rock is the best sample to attenuate gamma-rays among the selected rocks. Figure 4 displays the MFP of the natural rock at the 0.37–2.51 MEV region. One can see that the MFP values of the selected natural rock are very small at the low energies due to the photoelectric absorption. Then, the MFP increases gradually as energy increases, and this is attributed to multiple collisions of Compton scattering. Moreover, the sandstone sample has the highest values of MFP, while the limestone sample has the lowest values of MFP; thus, the photons can be attenuated swiftly in the limestone sample. The radiation shielding properties of the natural rock selected in this study were compared with some radiation shielding materials in terms of HVL at 0.662 MeV (in Figure 5) and at 2.51 MeV (in Figure 6). These figures demonstrate the potential use of the selected natural rocks in radiation applications as superior shielding materials. Clearly, the HVL of the natural rocks is smaller than that of ordinary concrete which is used as the conventional shield against ionizing radiation.

## 4. Conclusions

In the current investigation, we have examined the radiation shielding characteristics of some natural rocks, including olivine basalt, jet black granite, limestone, sandstone, and dolerite. The μ/ρ values of these rocks were obtained via EPICS2017 and the obtained results were verified via XCOM software. The HVL, MFP, and Z_eff_ were calculated for all the selected rocks. The maximum μ/ρ values of the natural rocks were observed at 0.37 MeV. At this energy, the Z_eff_ values of the natural rocks were 16.23, 16.97, 17.28, 10.43, and 16.65 for olivine basalt, jet black granite, limestone, sandstone, and dolerite, respectively. The radiation shielding characteristics of the studied rocks are found to be better than those of various traditional concretes, and very close to those of commercial glasses. Therefore, the natural rocks can be used as superior, economic, and environmentally friendly shields for radiation shielding applications.

## Figures and Tables

**Table 1:** Chemical composition and density of the selected natural rock.

Rock Type	Wt. Fraction of Elements in Samples											Density g/cm^3^
	O	Na	Mg	Al	Si	P	K	Ca	Ti	Mn	Fe	
Olivine Basalt	0.4419	0.0345	0.0261	0.0685	0.2336	0.0023	0.0079	0.0726	0.0167	0.0014	0.0945	2.72
Jet Black Granite	0.4352	0.0258	0.0215	0.0624	0.2210	0.0036	0.0092	0.0693	0.0232	0.0016	0.1272	2.64
Limestone	0.3734	0.0001	0.0065	0.0178	0.1509	0.0006	0.0053	0.4105	0.0024	0.0008	0.0317	2.73
Sandstone	0.5265	0.0001	0.0001	0.0281	0.4370	0.0002	0.0001	0.0021	0.0006	0.0005	0.0047	2.51
Dolerite	0.4399	0.0298	0.0229	0.0646	0.2298	0.0015	0.0026	0.0707	0.0226	0.0017	0.1139	2.65

**Table 2:** Mass attenuation coefficient (μ/ρ) of the olivine basalt, jet black granite, and limestone natural rocks obtained by the EPICS2017 and XCOM programmes at different photons energies.

Energy (MeV)	Olivine Basalt			Jet Black Granite			Limestone		
	EPICS2017	XCOM	Dev.%	EPICS2017	XCOM	Dev.%	EPICS2017	XCOM	Dev.%
0.37	0.0985	0.1001	1.5984	0.0984	0.1001	1.6983	0.0997	0.1014	1.6765
0.51	0.0859	0.0859	0.0001	0.0858	0.0858	0.0001	0.0868	0.0868	0.0001
0.66	0.0766	0.0765	0.1307	0.0764	0.0764	0.0001	0.0773	0.0773	0.0001
10.83	0.0688	0.0691	0.4342	0.0687	0.0689	0.2903	0.0695	0.0697	0.2869
1.17	0.0581	0.0582	0.1718	0.0580	0.0581	0.1721	0.0587	0.0588	0.1701
1.28	0.0556	0.0556	0.0001	0.0554	0.0555	0.1802	0.0561	0.0562	0.1779
1.33	0.0544	0.0545	0.1835	0.0543	0.0544	0.1838	0.0550	0.0551	0.1815
2.51	0.0394	0.0395	0.2532	0.0394	0.0395	0.2532	0.0400	0.0401	0.2494

**Table 3:** Mass attenuation coefficient (μ/ρ) of the sandstone and dolerite natural rocks obtained by the EPICS2017 and XCOM programmes at different photons energies.

Photon Energy (MeV)	Sandstone			Dolerite		
	EPICS2017	XCOM	Dev.%	EPICS2017	XCOM	Dev.%
0.37	0.0988	0.1004	1.5936	0.0989	0.1001	1.1988
0.51	0.0865	0.0865	0.0001	0.0858	0.0858	0.0001
0.66	0.0772	0.0772	0.0001	0.0764	0.0765	0.1307
0.83	0.0695	0.0697	0.2869	0.0693	0.0690	0.4348
1.17	0.0587	0.0588	0.1701	0.0587	0.0582	0.8591
1.28	0.0561	0.0562	0.1779	0.0560	0.0556	0.7194
1.33	0.0550	0.0551	0.1815	0.0548	0.0545	0.5505
2.51	0.0396	0.0397	0.2519	0.0397	0.0395	0.5063

## Data Availability

The data presented in this study are available on request from the corresponding author.
